# 2- and 6-*O*-sulfated proteoglycans have distinct and complementary roles in cranial axon guidance and motor neuron migration

**DOI:** 10.1242/dev.126854

**Published:** 2016-06-01

**Authors:** Miguel Tillo, Camille Charoy, Quenten Schwarz, Charlotte H. Maden, Kathryn Davidson, Alessandro Fantin, Christiana Ruhrberg

**Affiliations:** 1 UCL Institute of Ophthalmology, University College London, 11-43 Bath Street, London EC1V 9EL, UK; 2 Yale Cardiovascular Research Centre, Yale University, New Haven, CT 06511, USA

**Keywords:** Fibroblast growth factor (FGF), Vascular endothelial growth factor (VEGF), Heparan sulfate proteoglycan (HSPG), Sulfotransferase, Neuron migration, Axon guidance

## Abstract

The correct migration and axon extension of neurons in the developing nervous system is essential for the appropriate wiring and function of neural networks. Here, we report that *O*-sulfotransferases, a class of enzymes that modify heparan sulfate proteoglycans (HSPGs), are essential to regulate neuronal migration and axon development. We show that the 6-*O*-sulfotransferases HS6ST1 and HS6ST2 are essential for cranial axon patterning, whilst the 2-*O*-sulfotransferase HS2ST (also known as HS2ST1) is important to regulate the migration of facial branchiomotor (FBM) neurons in the hindbrain. We have also investigated how HS2ST interacts with other signals in the hindbrain and show that fibroblast growth factor (FGF) signalling regulates FBM neuron migration in an HS2ST-dependent manner.

## Abstract (precis)

**Summary:** 2-*O*-sulfated proteoglycans are essential for cranial motor neuron migration, whereas 6-*O*-sulfated proteoglycans regulate cranial axon guidance.

## INTRODUCTION

Various developmental processes require heparan sulfate proteoglycans (HSPGs), extracellular matrix proteins with covalently linked polysaccharide side chains that are polymerised by exotosin enzymes and further modified by sulfation, epimerisation or deacetylation to generate vast structural and functional heterogeneity (Kreuger and Kjellen, 2012). Two families of modifying enzymes, the 2- and 6-*O*-sulfotransferases, have previously been shown to regulate axon guidance in the *Xenopus* and mouse brain (Irie et al., 2002; Pratt et al., 2006; Clegg et al., 2014). Both enzymes also control neuronal migration in *Caenorhabditis* *elegans* (Kinnunen et al., 2005). However, it is not known whether these enzymes regulate neuronal migration in the vertebrate brain.

The proper positioning of neurons in the developing brain and the correct extension of their neurites to suitable targets are fundamental processes for the appropriate wiring and therefore function of the nervous system. The facial (VIIth) and trigeminal (Vth) cranial nerves are widely used models to define molecular mechanisms of axon guidance (Chandrasekhar, 2004; Wanner et al., 2013). Whilst the trigeminal motor neurons arise in hindbrain rhombomere 2 (r2), the facial branchiomotor (FBM) neurons arise in r4. Both types of neurons extend their axons dorsally out of the hindbrain into the branchial arches in stereotypical patterns that can be visualised by whole-mount immunostaining. Mouse FBM neurons are useful to study neuronal migration, because ISL1 labelling can be used to observe their cell bodies as they migrate caudally from r4 through r5 before turning ventrally to reach their final position in r6 (e.g. Schwarz et al., 2004). Several molecular signals cooperatively control FBM neuron migration (Chandrasekhar, 2004; Wanner et al., 2013), for example, the secreted extracellular matrix protein reelin and components of the WNT planar cell polarity (PCP) pathway (Rossel et al., 2005; Vivancos et al., 2009; Qu et al., 2010). The vascular endothelial growth factor (VEGF-A; short, VEGF) also guides FBM neuron migration through its receptor neuropilin 1 (NRP1); interestingly, NRP1 also serves as a receptor for repulsive SEMA3A signals during FBM axon guidance (Taniguchi et al., 1997; Schwarz et al., 2004).

*O*-sulfotransferases have previously been implicated in VEGF, fibroblast growth factor (FGF) and WNT signalling (e.g. Ai et al., 2003; Kamimura et al., 2006; Mitsi et al., 2006). The 6-*O*-sulfotransferases HS6ST1 and HS6ST2 promote the formation of VEGF/VEGFR and FGF/FGFR signalling complexes in vascular endothelial cells (Ferreras et al., 2012). HS6ST2 is also required for VEGF signalling during zebrafish vascular development (Chen et al., 2005). Moreover, FGFs interact with *O*-sulfated HSPGs during lacrimal gland and tracheal development (Kamimura et al., 2006; Qu et al., 2011). WNT signalling is regulated by 2-*O*-sulfotransferase (HS2ST, also known as HS2ST1) during zebrafish epiboly and by HS6STs for muscle development (Bink et al., 2003; Cadwalader et al., 2012), suggesting that the role of *O*-sulfated HSPGs is context-specific.

Here, we show that mice lacking HS6ST1 and HS6ST2 have defective axon extension of specific branches of the Vth and VIIth cranial nerves, but normal FBM neuron migration. Conversely, mice lacking HS2ST showed normal cranial axon patterning, but had FBM neuron migration defects similar to those seen in mice lacking VEGF signalling through NRP1. Surprisingly, however, HS2ST was not required for VEGF signalling in these neurons. Instead, HS2ST enabled FGF-mediated FBM neuron migration in hindbrain explants. Moreover, the expression of *Erm*, a known FGF target, was altered in these mutants. Thus, our study has revealed a role for HSPG-mediated FGF signalling in neuronal migration and demonstrated distinct and complementary roles for 2- and 6-*O*-sulfotransferases in cranial nerve development.

## RESULTS AND DISCUSSION

### *Hs6st1* and *Hs6st2* cooperate in cranial axon guidance, but are dispensable for FBM neuron migration

**Fig. 1.**
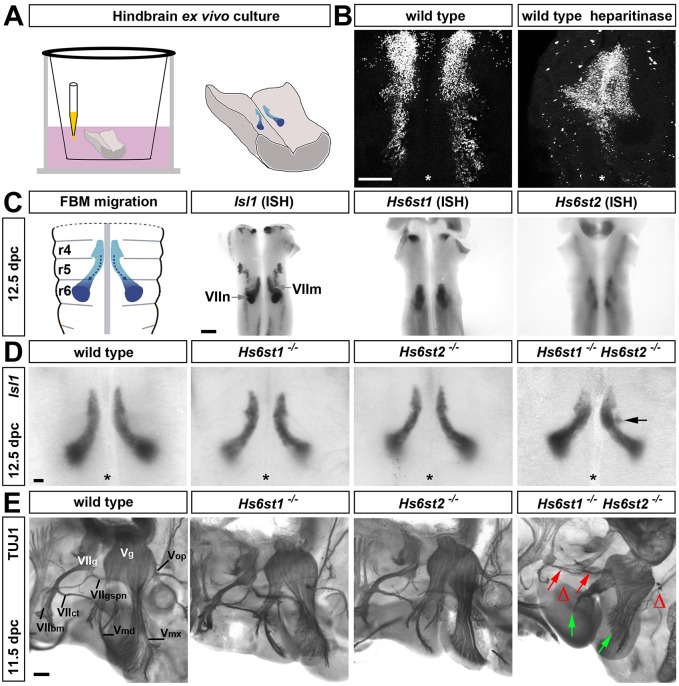
***Hs6st1* and *Hs6st2* regulate cranial axon guidance, but not FBM neuron migration.** (A) 11.5 dpc hindbrain explants were treated with inhibitors or implanted with beads containing growth factors. (B) Hindbrain explants (*n*=4) were treated with vehicle or heparitinase and immunostained for ISL1. Asterisks indicate the midline. (C) Schematic representation of FBM neuron migration and ISH of 12.5 dpc hindbrains to reveal *Hs6st1* and *Hs6st2* expression relative to *Isl1-*positive migrating FBM neurons (VII_m_) and the paired facial motor nuclei (VII_n_). (D) *Isl1* ISH of *Hs6st1^−/−^* (*n*=3), *Hs6st2^−/−^* (*n*=10) , *Hs6st1*^*−/−*^*;Hs6st2*^*−/−*^ (minor defects in 2/5 mutants examined) and wild-type (*n*=6) hindbrains; the arrow indicates a minor defect in FBM neuron migration. (E) Lateral view of 11.5 dpc *Hs6st1^−/−^*, *Hs6st2^−/−^* and *Hs6st1^−/−^;Hs6st2^−/−^* mutant (*n*=3 each) and wild-type (*n*=5) heads after immunolabelling for TUJ1. Green arrows indicate delayed extension of the mandibular (V_md_) and maxillary (V_mx_) nerves from the trigeminal ganglion (V_g_), red arrows indicate abnormal chorda tympani (VII_ct_) and facial branchiomotor nerve (VII_bm_) branches from the facial ganglion (VII_g_) and open triangles the lack of ophthalmic (V_op_) and greater superficial petrosal (VII_gspn_) nerves. Scale bars: 200 μm in B,C,E; 100 μm in D.

To examine whether HSPGs regulate FBM neuron migration, we used an *ex vivo* hindbrain culture assay (Fig. 1A). In this model, FBM neurons migrate rostrally from their site of origin in r4 towards r6, similar to the migration they undergo *in vivo* (Tillo et al., 2014). The inclusion of heparitinase in the culture medium to remove heparan sulfate side chains from HSPGs (Linhardt et al., 1990) prevented FBM neuron migration beyond r5 (Fig. 1B). This observation suggested that HSPGs are important for FBM neuron migration. As HS6ST2 is also required for VEGF signalling during zebrafish vascular development, we examined the expression of 6-*O*-sulfotransferases during FBM migration. *In situ* hybridisation (ISH) at 12.5 days post coitum (dpc) showed that *Hs6st3* was not obviously expressed in the hindbrain at this stage (data not shown), but *Hs6st1* and *Hs6st2* were expressed in the r5/6-derived hindbrain territories, through which the *Isl1*-expressing FBM neurons migrate (Fig. 1C). However, *Hs6st1^−/−^* and *Hst6s2^−/−^* single mutants had normal FBM neuron migration, and two out of five double mutant *Hs6st1^−/−^ Hs6st2^−/−^* hindbrains examined showed only minor displacement of some neurons (Fig. 1D). TUJ1 whole-mount immunostaining of *Hs6st1^−/−^* and *Hst6s2^−/−^* heads showed normal guidance of facial nerve axons, including those in the facial branchiomotor nerve (VII_bm_), the chorda tympani (VII_ct_) and the greater superficial petrosal nerve (VII_gspn_) (Fig. 1E). Trigeminal axons also seemed normal in these single mutants, including those of the mandibular (V_md_), maxillary (V_mx_) and ophthalmic (V_op_) nerves. However, the VII_bm_, VII_ct_ and VII_gspn_ and V_mx_ nerves did not extend normally and the V_md_ and V_op_ nerves were absent in three out of three *Hs6st1^−/−^ Hs6st2^−/−^* double mutants (Fig. 1E).

These results demonstrate that 6-*O*-sulfotransferases are dispensable for FBM neuron migration, but essential for cranial nerve axon guidance.

### 6-*O*-sulfotransferases modulate trigeminal axon extension *in vitro*

The trigeminal axon defects of *Hs6st1^−/−^ Hs6st2^−/−^* mutants resembled those of *Slit* mutants (Ma and Tessier-Lavigne, 2007). To investigate whether these 6*-O*-sulfotransferases are required for SLIT proteins to modulate trigeminal axon extension, we explanted trigeminal ganglia to observe neurite extension in the absence or presence of SLIT proteins (Fig. S1). In culture media lacking exogenous SLITs, 6*-O*-sulfotransferase deficiency increased the number of neurites (Fig. S1A,B) and the maximal distance of neurite extension from the explant (Fig. S1A,C). Moreover, compound mutant explants extended unusually long and thick neurites with a non-linear growth pattern that were rarely present in wild-type and single mutant explants (Fig. S1A). These findings corroborate that 6*-O*-sulfotransferases modulate trigeminal axon growth. However, 6*-O*-sulfotransferases were inhibitory in this assay, even though they are required for axon path-finding *in vivo* (Fig. 1E). This observation raises the possibility that explant cultures lack factors that alter 6*-O*-sulfotransferase-dependent processes from inhibitory to growth-promoting. Recombinant SLIT2, but not SLIT1, reduced neurite extension from wild-type explants (Fig. S1D,E). SLIT2 also reduced neurite extension from mutant explants to a similar extent as from wild-type explants (Fig. S1F,G). Moreover, SLIT2 did not decrease the formation of aberrant long and thick neurites in compound mutant explants (Fig. S1F). Thus, SLIT2 and 6*-O*-sulfotransferases regulate neurite extension independently of each other, and HS6ST1 and HS6ST2 promote cranial axon guidance in pathways that remain to be identified.

### *Hs2st* is essential for FBM neuron migration, but dispensable for cranial axon guidance

**Fig. 2.**
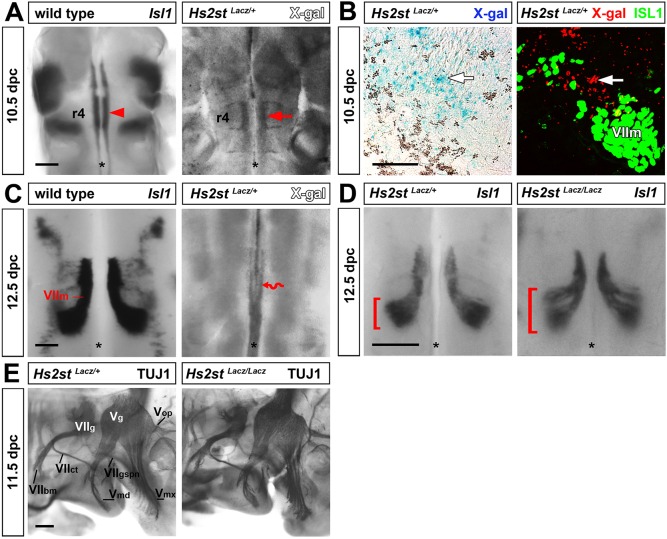
***Hs2st* is expressed near FBM neurons and is essential for their appropriate migration*.*** (A) Whole-mount X-gal staining of 10.5 dpc *Hs2st^Lacz/+^* hindbrains (*n*=5) shows *Hs2st* expression in r4 (arrow) adjacent to the motor column, visualised by *Isl1* ISH (arrowhead). (B) Transverse 10.5 dpc *Hs2st^Lacz/+^* sections through r4 level were X-gal stained and then immunostained for ISL1 to demonstrate *Hs2st* expression adjacent to FBM neurons (VII_m_; the X-gal stain was pseudocoloured red in the right-hand panel). The arrow indicates *Hs2st* expression. (C) Whole-mount X-gal staining of 12.5 dpc *Hs2st^Lacz/+^* hindbrains (*n*=3) shows *Hs2st* expression in the midline area (red wavy arrow). (D) *Isl1* whole-mount ISH of *Hs2st^Lacz/Lacz^* and wild-type hindbrains (*n*=6 each); brackets indicate the width of the FBM neuron stream. (E) Lateral views of 11.5 dpc *Hs2st^Lacz/Lacz^* heads and controls, immunolabelled for TUJ1 (*n*=3 each). The V_md_, V_mx_ and V_op_ nerves extend from the trigeminal ganglion (V_g_), and the VII_ct_, VII_bm_ and VII_gspn_ nerves from the facial ganglion (VII_g_) in both genotypes. Scale bars: 200 μm in A,C,E; 400 μm in D; 50 μm in B.

To determine the expression pattern of HS2ST during hindbrain development, we used mice carrying an *Hs2st^Lacz^* knock-in allele that recapitulates endogenous *Hs2st* expression when visualised as β-galactosidase-mediated X-gal staining (Bullock et al., 1998). *Hs2st^Lacz/+^* hindbrains at 10.5 dpc showed prominent staining in r4, close to the domain in which FBM neurons differentiate (Fig. 2A). The *Hs2st* expression domain was adjacent to, but did not overlap with, the area that contains FBM neurons (Fig. 2B). By 12.5 dpc, *Hs2st* expression seemed to be restricted to the midline region (Fig. 2C). These expression patterns raise the possibility that HS2ST modifies proteoglycans that regulate FBM neuron development in *trans*. To determine whether HS2ST is required for FBM neuron migration, we examined *Hs2st*-null (*Hs2st^Lacz/Lacz^*) hindbrains by *Isl1* ISH. We observed splitting of the migratory stream in all mutants examined (Fig. 2D). By contrast, whole-mount TUJ1 immunolabelling showed normal axon extension of all facial and trigeminal nerve branches in mutants (Fig. 2E). These results suggest that 2-*O*-sulfotransferase is important for FBM neuron migration, but not cranial axon guidance.

### *Hs2st* is dispensable for VEGF-mediated FBM neuron migration

**Fig. 3.**
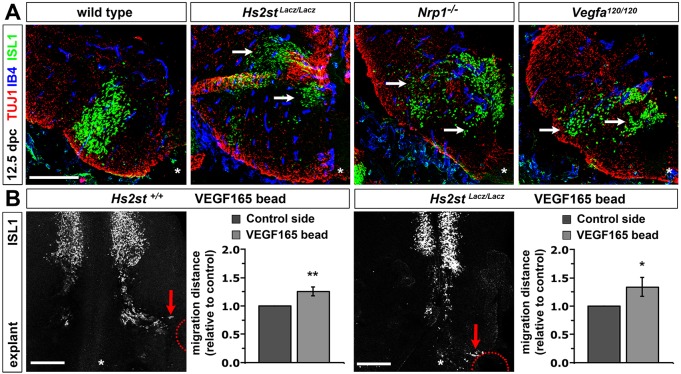
**VEGF does not require HS2ST to control FBM neuron migration.** (A) Transverse sections through 12.5 dpc r6, immunolabelled for ISL1, TUJ1 and the vascular marker IB4; arrows indicate similar stream splitting of FBM neurons in *Hs2st^Lacz/Lacz^*, *Nrp1^−/−^* and *Vegfa^120/120^* mice (*n*=3 each). (B) Hindbrain explants containing VEGF_165_ beads (position indicated with red circles) were immunostained for ISL1; red arrows indicate the leading edge of FBM neurons migrating towards the beads. The distance migrated on the implanted relative to the control side of each hindbrain is shown as mean±s.e.m. *Hs2st^+/+^*, *n*=15; *Hs2st^Lacz/Lacz^*, *n*=7. *P<0.05, **P<0.01, paired *t*-test. Scale bars: 200 μm.

HSPGs interact with VEGF and its receptor NRP1 (Sarrazin et al., 2011). We therefore asked if loss of 2-*O*-sulfated HSPGs caused an FBM neuron defect similar to that caused by loss of VEGF signalling through NRP1 (Schwarz et al., 2004). Comparable to *Nrp1*-null mice and *Vegfa^120/120^* mice lacking NRP1-binding VEGF (Schwarz et al., 2004), migrating FBM neurons split into two main streams in *Hs2st*-null hindbrains (Fig. 3A). To examine whether HS2ST-modified HSPGs were required for VEGF-induced FBM neuron migration, we implanted VEGF_165_-coated heparin beads into *Hs2st*-null and littermate control hindbrain explants. As previously shown (Schwarz et al., 2004), FBM neurons migrated towards the beads in controls (Fig. 3B). Unexpectedly, these neurons also migrated towards the beads in *Hs2st*-null hindbrains (Fig. 3B). Quantification confirmed that VEGF_165_ beads promoted FBM neuron migration in both genotypes (Fig. 3B). Despite the similar *in vivo* phenotypes of *Hs2st*-null and *Vegfa^120/120^* mice, HS2ST-modified HSPGs are therefore not required for VEGF-induced FBM neuron migration.

### FGF can promote FBM neuron migration in an HS2ST-dependent fashion

**Fig. 4.**
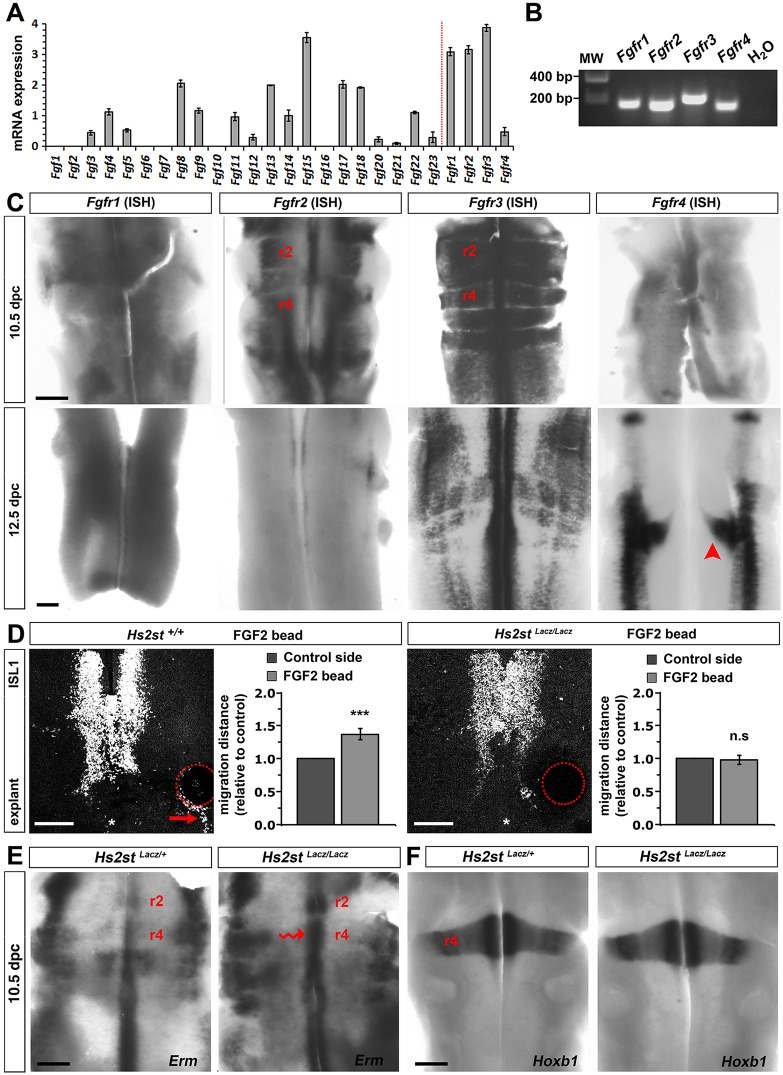
**FGF controls FBM neuron migration and requires Hs2st.** (A,B) Microarray (A) and RT-PCR (B) analysis of *Fgf* and *Fgfr* expression in 11.5 dpc hindbrains. Data in A are expressed as the mean±s.e.m. of the optical density for each probe set after normalisation and transformation to a log2 scale; *n*=3 wild-type hindbrains. (B) *Fgfr1* (148 bp), *Fgfr2* (142 bp), *Fgfr3* (197 bp) and *Fgfr4* (141 bp) amplicons after gel electrophoresis; MW, molecular weight standard. (C) Whole-mount ISH of *Fgfr* genes in 10.5 and 12.5 dpc hindbrains; the red arrowhead indicates *Fgfr4* expression in migrating FBM neurons. (D) *Hs2st^Lacz/Lacz^* and control explants containing FGF2 beads (indicated with red circles) were immunostained for ISL1; red arrow indicates the leading edge of FBM neurons migrating towards the beads. The distance migrated on the implanted relative to the control side of each hindbrain is shown as mean±s.e.m. *Hs2st^+/+^*, *n*=16; *Hs2st^Lacz/Lacz^*, *n*=5. ***P<0.001, n.s. not significant, paired *t*-test. (E) Whole-mount *Erm* ISH of 10.5 dpc *Hs2st*-null and wild-type hindbrains (*n*=3 each); the wavy red arrow indicates *Erm* upregulation in mutant compared with control r4. (F) Whole-mount *Hoxb1* ISH at 10.5 dpc shows normal r4 specification (*n*=2 each). Scale bars: 200 μm.

HSPGs act as FGF co-receptors and regulate the distribution and degradation of FGFs (Hacker et al., 2005). Microarray analysis showed that FGF3-5, 8, 9, 11-15 and 17-23 as well as the four FGF receptors (*Fgfr1*-*4*) were expressed in 11.5 dpc hindbrains (Fig. 4A). Reverse transcription PCR (RT-PCR) confirmed *Fgfr1*-*4* expression in 11.5 dpc hindbrains (Fig. 4B). ISH showed that *Fgfr1*-*4* were expressed widely at 10.5 dpc, the onset of FBM neuron migration, whilst at 12.5 dpc, *Fgfr1*-*3* were expressed near the midline and *Fgfr4* was expressed by FBM neurons when they reached r6 (Fig. 4C). To examine whether FBM neurons can in principle respond to FGF ligands, we performed hindbrain explants with FGF-coated beads. For these experiments, we used FGF2, because it is known to bind all four FGF receptors and requires HSPGs for appropriate signalling (Matsuo and Kimura-Yoshida, 2013). FBM neurons in wild-type hindbrains were strongly attracted by FGF2 beads, but attraction was abolished in *Hs2st*-null hindbrains (Fig. 4D). Quantification confirmed significantly impaired FGF2-induced FBM neuron migration in the mutants (Fig. 4D). These observations raise the possibility that FGFs promote FBM neuron migration in a mechanism that requires 2-*O*-sulfated HSPGs. However, further experiments are required to define which endogenous FGF ligand or receptor might regulate FBM neuron development *in vivo*.

As *Hs2st* is prominently expressed in r4 at 10.5 dpc, we considered the possibility that HSPGs modulate FGF-induced signalling pathways in this rhombomere to regulate FBM neuron migration. We observed that *Erm* (also known as *Etv5*), a member of the ETS transcription factor family that is positively regulated by FGF (Raible and Brand, 2001; Firnberg and Neubuser, 2002), was expressed in r6-r8 along the midline at 10.5 dpc (Fig. 4E). Notably, 10.5 dpc *Hs2st*-null hindbrains upregulated *Erm* expression in the midline region of r2 and r4, the rhombomeres that give rise to the trigeminal branchiomotor and FBM neurons, respectively (Fig. 4E). This finding agrees with prior observations that HSPGs can spatially restrict the diffusion or degradation of FGF ligands in the zebrafish embryo to modulate FGF signalling (Venero Galanternik et al., 2015). In contrast to *Erm*, the master regulator of r4, *Hoxb1*, was expressed normally in 10.5 dpc *Hs2st*-null hindbrains (Fig. 4F). This observation suggests that r4 is specified normally in *Hs2st*-null mutants and agrees with *Erm* upregulation resulting from HS2ST-dependent local alterations in FGF signalling. Together, our findings are consistent with a role for HS2ST in FGF-mediated neuronal guidance and/or hindbrain patterning. Future studies might investigate whether HS2ST also regulates other molecules that control FBM migration non-cell autonomously, such as cadherins and PCP pathway components (Vivancos et al., 2009; Qu et al., 2010; Stockinger et al., 2011; Zakaria et al., 2014).

### Conclusion

*Hs6st1* and *Hs6st2* synergise to regulate cranial axon guidance whilst *Hs2st* promotes FBM neuron migration. Thus, HS6ST1/2 and HS2ST have distinct, but complementary, roles in cranial nerve development.

## MATERIALS AND METHODS

### Animals

Animal procedures were performed in accordance with institutional guidelines under the authority of a UK Home Office project licence. Mice were paired in the evening, and the morning of vaginal plug formation counted as 0.5 dpc. We used *Hs2st^Lacz/+^* (Bullock et al., 1998), *Hs6st1^+/−^* (Leighton et al., 2001; Mitchell et al., 2001), *Hs6st2^+/−^* (Qu et al., 2011) and *Vegfa^120/+^* (Carmeliet et al., 1999) mice on a C57/Bl6 background and *Nrp1^+/−^* (Kitsukawa et al., 1997) mice on a CD1 background.

### Microarray and RT-PCR analysis

Microarray analysis was performed with the GeneChip Mouse 1.0 ST Array (Affimetrix) on 11.5 dpc *Spi1^−^*^/−^ and *Spi1*^+/+^ hindbrains (*n*=3 each); expression was measured as optical density for each probe set, values were normalised by robust multi-array analysis and transformed to a log2 scale (Irizarry et al., 2003); the *Spi1* and the SPI1 (PU.1)-dependent *Csf1r* values in *Spi1^−^*^/−^ samples were defined as background noise and subtracted from the value of each gene. In some experiments, RNA was reverse transcribed using Superscript III (Thermo Fisher) and cDNA amplified by PCR using MegaMix (Microzone) and the following oligonucleotides (Sigma): *Fgfr1*: 5′-AACCTCTAACCGCAGAAC-3′ and 5′-GAGACTCCACTTCCACAG-3′; *Fgfr2*: 5′-AATCTCCCAACCAGAAGCGTA-3′ and 5′-CTCCCCAATAAGCACTGTCCT-3′; *Fgfr3*: 5′-GGAGGACGTGGCTGAAGAC-3′ and 5′-GGAGCTTGATGCCCCCAAT-3′; *Fgfr4*: 5′-ATGACCGTCGTACACAATCTTAC-3′ and 5′-TGTCCAGTAGGGTGCTTGC-3′.

### Immunolabelling and *in situ* hybridisation

Immunolabelling was performed as described (Vieira et al., 2007) using rabbit anti-mouse TUJ1 (1:250; MRB-435P, BioLegend) and mouse anti-rat ISL1 (1:100; 39.4D5, DSHB) followed by Alexa Fluor 594-conjugated rabbit anti-goat Fab (1:200; A21223, Thermo Fisher) and Alexa Fluor 488-conjugated rabbit anti-mouse Fab (1:200; A11017, Thermo Fisher) or horseradish-peroxidase-conjugated goat anti-rabbit antibody (1:200; P0448, DAKO). To detect blood vessels, we used biotinylated isolectin B4 (1:500; L2140, Sigma) followed by Alexa Fluor 633-conjugated streptavidin (1:200; S-21375, Thermo Fisher). We used digoxigenin-labelled RNA probes for *Isl1* (Schwarz et al., 2004), *Hoxb1* (Gavalas et al., 2003), *Hs6st1*, *Hs6st2* (Sedita et al., 2004), *Erm* (Trokovic et al., 2005) and *Fgfr1-4* (Pringle et al., 2003; Trokovic et al., 2003).

### Explant culture

E11.5 hindbrain explants were cultured as described (Tillo et al., 2014). Affi-Gel heparin beads (Bio-Rad) were soaked overnight in 100 ng/ml VEGF_165_ (PreproTech) or 10 ng/ml FGF2 (Sigma) before implantation. In some experiments, explants were treated with 8.25 mU/ml heparitinase from *Flavobacterium heparinum* (Seikagaku-Kogyo) in PBS. FBM neuron migration was measured with ImageJ (NIH, Bethesda) as the distance from r5 to the leading group of cells in r6 and normalised to the unimplanted side of each hindbrain. E12.5 trigeminal ganglia were cultured on coverslips coated in poly-L-ornithine and mouse laminin (Sigma) in Neurobasal media containing N2 and B27 (Thermo Fisher) and 4% methylcellulose for 40 h and then fixed in 4% formaldehyde for immunostaining with TUJ1 (Graef et al., 2003). In some experiments, explants were treated with 200 ng/ml recombinant SLIT1 and/or SLIT2 (R&D Systems) at 2, 18 and 24 h. Quantification of neurite extension was performed with the NeuriteJ plugin of ImageJ.

## References

[DEV126854C1] Ai X., Do A.-T., Lozynska O., Kusche-Gullberg M., Lindahl U. and Emerson C. P. Jr (2003). QSulf1 remodels the 6-O sulfation states of cell surface heparan sulfate proteoglycans to promote Wnt signaling. *J. Cell Biol.* 162, 341-351. 10.1083/jcb.200212083 12860968 PMC2172803

[DEV126854C2] Bink R. J., Habuchi H., Lele Z., Dolk E., Joore J., Rauch G.-J., Geisler R., Wilson S. W., den Hertog J., Kimata K. et al. (2003). Heparan sulfate 6-o-sulfotransferase is essential for muscle development in zebrafish. *J. Biol. Chem.* 278, 31118-31127. 10.1074/jbc.M213124200 12782624

[DEV126854C3] Bullock S. L., Fletcher J. M., Beddington R. S. P. and Wilson V. A. (1998). Renal agenesis in mice homozygous for a gene trap mutation in the gene encoding heparan sulfate 2-sulfotransferase. *Genes Dev.* 12, 1894-1906. 10.1101/gad.12.12.1894 9637690 PMC316906

[DEV126854C4] Cadwalader E. L., Condic M. L. and Yost H. J. (2012). 2-O-sulfotransferase regulates Wnt signaling, cell adhesion and cell cycle during zebrafish epiboly. *Development* 139, 1296-1305. 10.1242/dev.078238 22357927 PMC3294434

[DEV126854C5] Carmeliet P., Ng Y.-S., Nuyens D., Theilmeier G., Brusselmans K., Cornelissen I., Ehler E., Kakkar V. V., Stalmans I., Mattot V. et al. (1999). Impaired myocardial angiogenesis and ischemic cardiomyopathy in mice lacking the vascular endothelial growth factor isoforms VEGF164 and VEGF188. *Nat. Med.* 5, 495-502. 10.1038/8379 10229225

[DEV126854C6] Chandrasekhar A. (2004). Turning heads: development of vertebrate branchiomotor neurons. *Dev. Dyn.* 229, 143-161. 10.1002/dvdy.10444 14699587 PMC2219919

[DEV126854C7] Chen E., Stringer S. E., Rusch M. A., Selleck S. B. and Ekker S. C. (2005). A unique role for 6-O sulfation modification in zebrafish vascular development. *Dev. Biol.* 284, 364-376. 10.1016/j.ydbio.2005.05.032 16009360

[DEV126854C8] Clegg J. M., Conway C. D., Howe K. M., Price D. J., Mason J. O., Turnbull J. E., Basson M. A. and Pratt T. (2014). Heparan sulfotransferases Hs6st1 and Hs2st keep Erk in check for mouse corpus callosum development. *J. Neurosci.* 34, 2389-2401. 10.1523/JNEUROSCI.3157-13.2014 24501377 PMC3913879

[DEV126854C9] Ferreras C., Rushton G., Cole C. L., Babur M., Telfer B. A., van Kuppevelt T. H., Gardiner J. M., Williams K. J., Jayson G. C. and Avizienyte E. (2012). Endothelial heparan sulfate 6-O-sulfation levels regulate angiogenic responses of endothelial cells to fibroblast growth factor 2 and vascular endothelial growth factor. *J. Biol. Chem.* 287, 36132-36146. 10.1074/jbc.M112.384875 22927437 PMC3476281

[DEV126854C10] Firnberg N. and Neubüser A. (2002). FGF signaling regulates expression of Tbx2, Erm, Pea3, and Pax3 in the early nasal region. *Dev. Biol.* 247, 237-250. 10.1006/dbio.2002.0696 12086464

[DEV126854C11] Gavalas A., Ruhrberg C., Livet J., Henderson C. E. and Krumlauf R. (2003). Neuronal defects in the hindbrain of Hoxa1, Hoxb1 and Hoxb2 mutants reflect regulatory interactions among these Hox genes. *Development* 130, 5663-5679. 10.1242/dev.00802 14522873

[DEV126854C12] Graef I. A., Wang F., Charron F., Chen L., Neilson J., Tessier-Lavigne M. and Crabtree G. R. (2003). Neurotrophins and netrins require calcineurin/NFAT signaling to stimulate outgrowth of embryonic axons. *Cell* 113, 657-670. 10.1016/S0092-8674(03)00390-8 12787506

[DEV126854C13] Häcker U., Nybakken K. and Perrimon N. (2005). Heparan sulphate proteoglycans: the sweet side of development. *Nat. Rev. Mol. Cell Biol.* 6, 530-541. 10.1038/nrm1681 16072037

[DEV126854C14] Irie A., Yates E. A., Turnbull J. E. and Holt C. E. (2002). Specific heparan sulfate structures involved in retinal axon targeting. *Development* 129, 61-70. 11782401 10.1242/dev.129.1.61

[DEV126854C15] Irizarry R. A., Bolstad B. M., Collin F., Cope L. M., Hobbs B. and Speed T. P. (2003). Summaries of Affymetrix GeneChip probe level data. *Nucleic Acids Res.* 31, e15 10.1093/nar/gng015 12582260 PMC150247

[DEV126854C16] Kamimura K., Koyama T., Habuchi H., Ueda R., Masu M., Kimata K. and Nakato H. (2006). Specific and flexible roles of heparan sulfate modifications in Drosophila FGF signaling. *J. Cell Biol.* 174, 773-778. 10.1083/jcb.200603129 16966419 PMC2064332

[DEV126854C17] Kinnunen T., Huang Z., Townsend J., Gatdula M. M., Brown J. R., Esko J. D. and Turnbull J. E. (2005). Heparan 2-O-sulfotransferase, hst-2, is essential for normal cell migration in Caenorhabditis elegans. *Proc. Natl. Acad. Sci. USA* 102, 1507-1512. 10.1073/pnas.0401591102 15671174 PMC547812

[DEV126854C18] Kitsukawa T., Shimizu M., Sanbo M., Hirata T., Taniguchi M., Bekku Y., Yagi T. and Fujisawa H. (1997). Neuropilin-semaphorin III/D-mediated chemorepulsive signals play a crucial role in peripheral nerve projection in mice. *Neuron* 19, 995-1005. 10.1016/S0896-6273(00)80392-X 9390514

[DEV126854C19] Kreuger J. and Kjellen L. (2012). Heparan sulfate biosynthesis: regulation and variability. *J. Histochem. Cytochem.* 60, 898-907. 10.1369/0022155412464972 23042481 PMC3527889

[DEV126854C20] Leighton P. A., Mitchell K. J., Goodrich L. V., Lu X., Pinson K., Scherz P., Skarnes W. C. and Tessier-Lavigne M. (2001). Defining brain wiring patterns and mechanisms through gene trapping in mice. *Nature* 410, 174-179. 10.1038/35065539 11242070

[DEV126854C21] Linhardt R. J., Turnbull J. E., Wang H. M., Loganathan D. and Gallagher J. T. (1990). Examination of the substrate specificity of heparin and heparan sulfate lyases. *Biochemistry* 29, 2611-2617. 10.1021/bi00462a026 2334685

[DEV126854C22] Ma L. and Tessier-Lavigne M. (2007). Dual branch-promoting and branch-repelling actions of Slit/Robo signaling on peripheral and central branches of developing sensory axons. *J. Neurosci.* 27, 6843-6851. 10.1523/JNEUROSCI.1479-07.2007 17581972 PMC6672698

[DEV126854C23] Matsuo I. and Kimura-Yoshida C. (2013). Extracellular modulation of Fibroblast Growth Factor signaling through heparan sulfate proteoglycans in mammalian development. *Curr. Opin. Genet. Dev.* 23, 399-407. 10.1016/j.gde.2013.02.004 23465883

[DEV126854C24] Mitchell K. J., Pinson K. I., Kelly O. G., Brennan J., Zupicich J., Scherz P., Leighton P. A., Goodrich L. V., Lu X., Avery B. J. et al. (2001). Functional analysis of secreted and transmembrane proteins critical to mouse development. *Nat. Genet.* 28, 241-249. 10.1038/90074 11431694

[DEV126854C25] Mitsi M., Hong Z., Costello C. E. and Nugent M. A. (2006). Heparin-mediated conformational changes in fibronectin expose vascular endothelial growth factor binding sites. *Biochemistry* 45, 10319-10328. 10.1021/bi060974p 16922507

[DEV126854C26] Pratt T., Conway C. D., Tian N. M. M.-L., Price D. J. and Mason J. O. (2006). Heparan sulphation patterns generated by specific heparan sulfotransferase enzymes direct distinct aspects of retinal axon guidance at the optic chiasm. *J. Neurosci.* 26, 6911-6923. 10.1523/JNEUROSCI.0505-06.2006 16807321 PMC6673919

[DEV126854C27] Pringle N. P., Yu W.-P., Howell M., Colvin J. S., Ornitz D. M. and Richardson W. D. (2003). Fgfr3 expression by astrocytes and their precursors: evidence that astrocytes and oligodendrocytes originate in distinct neuroepithelial domains. *Development* 130, 93-102. 10.1242/dev.00184 12441294

[DEV126854C28] Qu Y., Glasco D. M., Zhou L., Sawant A., Ravni A., Fritzsch B., Damrau C., Murdoch J. N., Evans S., Pfaff S. L. et al. (2010). Atypical cadherins Celsr1–3 differentially regulate migration of facial branchiomotor neurons in mice. *J. Neurosci.* 30, 9392-9401. 10.1523/JNEUROSCI.0124-10.2010 20631168 PMC3069688

[DEV126854C29] Qu X., Carbe C., Tao C., Powers A., Lawrence R., van Kuppevelt T. H., Cardoso W. V., Grobe K., Esko J. D. and Zhang X. (2011). Lacrimal gland development and Fgf10-Fgfr2b signaling are controlled by 2-O- and 6-O-sulfated heparan sulfate. *J. Biol. Chem.* 286, 14435-14444. 10.1074/jbc.M111.225003 21357686 PMC3077643

[DEV126854C30] Raible F. and Brand M. (2001). Tight transcriptional control of the ETS domain factors Erm and Pea3 by Fgf signaling during early zebrafish development. *Mech. Dev.* 107, 105-117. 10.1016/S0925-4773(01)00456-7 11520667

[DEV126854C31] Rossel M., Loulier K., Feuillet C., Alonso S. and Carroll P. (2005). Reelin signaling is necessary for a specific step in the migration of hindbrain efferent neurons. *Development* 132, 1175-1185. 10.1242/dev.01683 15703280

[DEV126854C32] Sarrazin S., Lamanna W. C. and Esko J. D. (2011). Heparan sulfate proteoglycans. *Cold Spring Harb. Perspec. Biol*. 3, a004952 10.1101/cshperspect.a004952 PMC3119907 21690215

[DEV126854C33] Schwarz Q., Gu C., Fujisawa H., Sabelko K., Gertsenstein M., Nagy A., Taniguchi M., Kolodkin A. L., Ginty D. D., Shima D. T. et al. (2004). Vascular endothelial growth factor controls neuronal migration and cooperates with Sema3A to pattern distinct compartments of the facial nerve. *Genes Dev.* 18, 2822-2834. 10.1101/gad.322904 15545635 PMC528901

[DEV126854C34] Sedita J., Izvolsky K. and Cardoso W. V. (2004). Differential expression of heparan sulfate 6-O-sulfotransferase isoforms in the mouse embryo suggests distinctive roles during organogenesis. *Dev. Dyn.* 231, 782-794. 10.1002/dvdy.20173 15499561

[DEV126854C35] Stockinger P., Maitre J.-L. and Heisenberg C.-P. (2011). Defective neuroepithelial cell cohesion affects tangential branchiomotor neuron migration in the zebrafish neural tube. *Development* 138, 4673-4683. 10.1242/dev.071233 21965614

[DEV126854C36] Taniguchi M., Yuasa S., Fujisawa H., Naruse I., Saga S., Mishina M. and Yagi T. (1997). Disruption of semaphorin III/D gene causes severe abnormality in peripheral nerve projection. *Neuron* 19, 519-530. 10.1016/S0896-6273(00)80368-2 9331345

[DEV126854C37] Tillo M., Schwarz Q. and Ruhrberg C. (2014). Mouse hindbrain ex vivo culture to study facial branchiomotor neuron migration. *J Vis. Exp.* 85, e51397 10.3791/51397 PMC4032788 24686480

[DEV126854C38] Trokovic R., Trokovic N., Hernesniemi S., Pirvola U., Vogt Weisenhorn D. M., Rossant J., McMahon A. P., Wurst W. and Partanen J. (2003). FGFR1 is independently required in both developing mid- and hindbrain for sustained response to isthmic signals. *EMBO J.* 22, 1811-1823. 10.1093/emboj/cdg169 12682014 PMC154461

[DEV126854C39] Trokovic R., Jukkola T., Saarimäki J., Peltopuro P., Naserke T., Vogt Weisenhorn D. M., Trokovic N., Wurst W. and Partanen J. (2005). Fgfr1-dependent boundary cells between developing mid- and hindbrain. *Dev. Biol.* 278, 428-439. 10.1016/j.ydbio.2004.11.024 15680361

[DEV126854C40] Venero Galanternik M., Kramer K. L. and Piotrowski T. (2015). Heparan sulfate proteoglycans regulate Fgf signaling and cell polarity during collective cell migration. *Cell Rep.* 10, 414-428. 10.1016/j.celrep.2014.12.043 PMC4531098 25600875

[DEV126854C41] Vieira J. M., Schwarz Q. and Ruhrberg C. (2007). Selective requirements for NRP1 ligands during neurovascular patterning. *Development* 134, 1833-1843. 10.1242/dev.002402 17428830 PMC2702678

[DEV126854C42] Vivancos V., Chen P., Spassky N., Qian D., Dabdoub A., Kelley M., Studer M. and Guthrie S. (2009). Wnt activity guides facial branchiomotor neuron migration, and involves the PCP pathway and JNK and ROCK kinases. *Neural Dev.* 4, 7 10.1186/1749-8104-4-7 19210786 PMC2654884

[DEV126854C43] Wanner S. J., Saeger I., Guthrie S. and Prince V. E. (2013). Facial motor neuron migration advances. *Curr. Opin Neurobiol.* 23, 943-950. 10.1016/j.conb.2013.09.001 24090878 PMC3852894

[DEV126854C44] Zakaria S., Mao Y., Kuta A., Ferreira de Sousa C., Gaufo G. O., McNeill H., Hindges R., Guthrie S., Irvine K. D. and Francis-West P. H. (2014). Regulation of neuronal migration by Dchs1-Fat4 planar cell polarity. *Curr. Biol.* 24, 1620-1627. 10.1016/j.cub.2014.05.067 24998526 PMC4193925

